# Three-year multi-mycotoxin analysis of South African commercial maize from three provinces

**DOI:** 10.3389/ffunb.2024.1426782

**Published:** 2024-12-02

**Authors:** Queenta Ngum Nji, Mulunda Mwanza

**Affiliations:** ^1^ Food Security and Safety Focus Area, Faculty of Natural and Agricultural Sciences, North-West University, Mmabatho, South Africa; ^2^ Department of Animal Health, Faculty of Natural and Agricultural Sciences, North-West University, Mmabatho, South Africa

**Keywords:** mycotoxins, LC-MS/MS, maize, food safety, South Africa

## Abstract

**Introduction:**

The Food and Agricultural Organization (FAO) reported that numerous diseases can be traced back to the consumption of unsafe food contaminated with mycotoxins. Mycotoxins are secondary metabolites produced by toxigenic filamentous fungi. Mycotoxins reported to be of socio-economic concerns include aflatoxins, fumonisins, zearalenone, ochratoxin A, and deoxynivalenol. These mycotoxins are frequent contaminants of maize especially in the face of climate change and global food insecurity. South Africa is a leading exporter of maize in Africa, hence, it is crucial to evaluate exposure risks with respect to mycotoxin contamination of maize for consumers’ safety.

**Materials and method:**

In total, 752 post-harvest maize samples collected from silos over a 3-year period were analysed using liquid chromatography with tandem mass spectrometry (LC-MS/MS) for the occurrence of mycotoxins.

**Results and discussion:**

The overall mean values for all the quantified mycotoxins were within the South Africa regulatory limit as well as the individual samples, apart from DON and FB mycotoxins with 5% and 1% samples, respectively, above the limit. Citrinin was quantified in South African commercial maize for the first time. The presence of major mycotoxins in South African commercial maize even within safety limits is of public health concern, hence, continuous monitoring and evaluation is recommended.

## 1 Introduction

Filamentous fungi, which are commonly found in agricultural produce such as peanuts, cereals, and especially maize, have the ability to produce toxic chemicals known as mycotoxins. Some of these fungi are capable of producing more than one mycotoxin and some mycotoxins are produced by more than one fungal species. Hence, co-occurrence with potential additive, antagonistic, or synergistic effects of mycotoxins is a common and natural phenomenon in foods and feeds compared to single mycotoxin contaminants (Xu et al., 2020). While mycotoxins occur more frequently in areas with a hot and humid climate that is favorable for the growth of some toxigenic fungi such as the *Aspergillus* species, other species such as *Fusarium* and their toxins [deoxynivalenol (DON), nivalenol, T-2 and HT-2 toxins, zearalenone (ZEA), and fumonisins (FBs)] are commonly found in temperate zones (Fapohunda and Adewunmi, 2019).

South Africa (SA) is classified as a semi-arid to arid country, characterized by variable climatic conditions ranging from a Mediterranean climate in the southwestern corner of the country to a temperate climate on the interior plateau, a subtropical climate in the eastern regions, subtropical, and arid in the western regions (Nji et al., 2022a; Nji et al., 2022b; Nji et al., 2022c). Among the more than 300 existing mycotoxins, aflatoxins (AFs), ochratoxins (OTs), zearalenone, fumonisins, trichothecenes, and some emerging mycotoxins (enniatin and beauvericin) are the most prevalent mycotoxins in food and feed with socio-economic concerns and hence are the most monitored (Carballo et al., 2021; Fan et al., 2019; Pallares et al., 2022; Zhang et al., 2023).

The main food groups affected by fungal metabolites are cereals, dried fruits, nuts, coffee, and spices (Arce-López et al., 2020), with maize being one of the major cereal grains that is consumed globally after wheat and rice. South Africa has been ranked as the ninth largest exporter of maize in the world, making it a leading exporter in Africa. In 2018, SA exported maize to 75 countries around the world (ITC, 2019). Maize and maize products with high consumption rates globally are considered one of the best substrates for fungi to grow and produce mycotoxins (Pereira et al., 2014). Mycotoxin contamination of crops has affected the exportation of these crops in many African countries such as Malawi, thereby causing heavy economic losses (Ambler et al., 2018; Edelman and Aberman, 2015). The estimation of losses linked to mycotoxin contamination of foods is often very hard to determine with certainty due to complex food systems and agricultural practices, but losses worth billions of dollars have been reported (Eskola et al., 2020b; Wu and Khlangwiset, 2010).

It is worth noting that mycotoxins exhibit different toxicity levels depending on the type of mycotoxin, individuals involved (age, health status), consumption dose, and duration of exposure to these toxins. Regulatory values differ from one type of mycotoxin to another and for different age groups. The incidence of liver cancers that are linked directly to the consumption of food contaminated with mycotoxins is on the rise globally (PACA, 2020). Due to their deleterious health effects on both humans and animals and their socio-economic impacts, mycotoxin contamination of food continues to draw global attention (Eskola et al., 2020a). A complete phytosanitary report on SA maize to ensure consumers’ safety is highly recommended to safeguard consumers, especially in the face of climate change. Sensitive and reliable methods are a necessity as mycotoxin toxicity occurs at very low concentrations, thus, liquid chromatography (LC) methodologies, coupled with tandem mass spectrometry (MS/MS) or high-resolution mass spectrometry (HRMS) are a common and reliable method for determining the exposure to mycotoxins (Al-Jaal et al., 2019; Owen et al., 2019). Due to global warming and climate change, maize can be highly contaminated with different mycotoxins above recommended safety levels. To verify this claim, the aims of this study were first to identify and quantify the mycotoxins present in South African commercial maize and second to evaluate whether the levels of mycotoxins were compliant with regulatory standards.

## 2 Materials and methods

### 2.1 Chemicals and standards

The chemicals and reagents used were obtained from Sigma-Aldrich (Vienna, Austria), Merck (Darmstadt, Germany), and VWR (Leuven, Belgium) and all were of analytical grade. Mycotoxin standards were purchased from different suppliers [Romer Labs^®^Inc. (Tulln, Austria), AnalytiCon Discovery (Potsdam, Germany), and Bio Australis (Smithfield, Australia)]. Purified water was obtained using reverse osmosis purification technology from Purite (Suez, UK) via LASEC, South Africa.

### 2.2 Sampling sites

**Figure 1:**
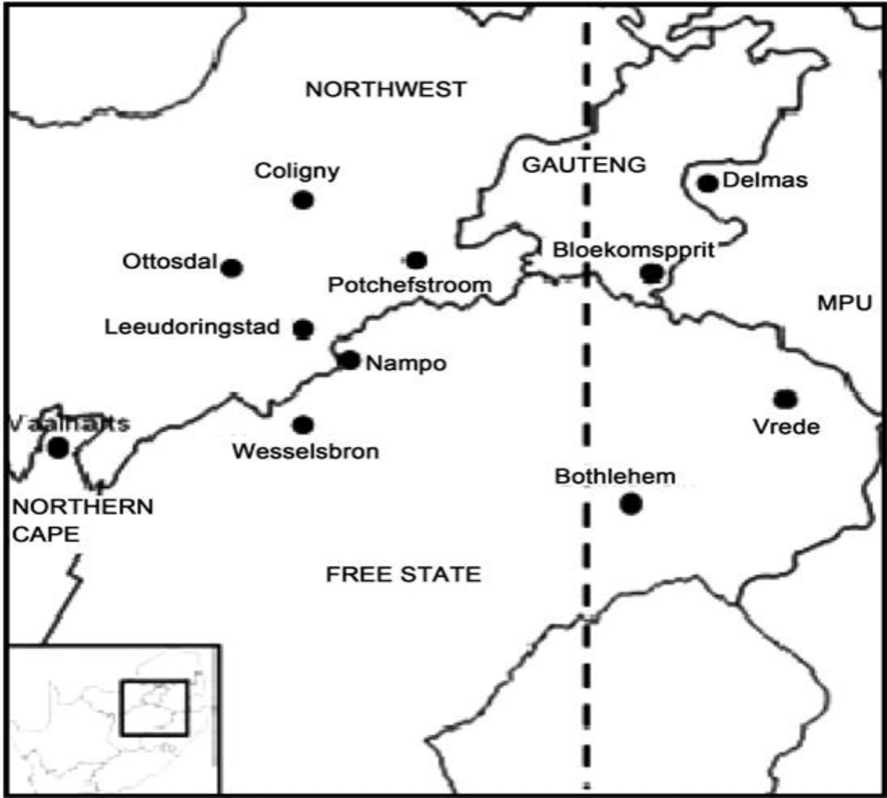
Map showing the areas in South Africa where maize samples were collected.

Experiments were conducted in three SA provinces, namely the Free State, North West, and Gauteng, which account for over 70% of the maize produced commercially (Saifaddin, 2021) (**Figure 1**). The Free State lies at latitude 28.4541°S and longitude 26.7968°E; the North West province lies at latitude 26.6639°S and longitude 25.2838°E; and Gauteng lies at latitude 26.1614°S and longitude 28.6442°E. Generally, South Africa is a semi-arid to arid country, and because of weather extremes, is characterized by a highly variable climate with constrained water resources. The country’s climatic conditions range from a Mediterranean climate in the southwestern corner of the country to a temperate climate in the interior plateau, subtropical towards the east, and arid towards the west. South Africa’s average annual rainfall is approximately 450 mm/year (Botai et al., 2018) and varies significantly from west to east and year on year.

### 2.3 Sampling and sample preparation

In total, 752 maize samples were randomly collected from selected silos in three provinces in SA over a period of 3 years (2017-2019). Approximately 1 kg of maize samples was collected with at least 10 incremental samples of 100 g each according to EC 401/2006 in sterile zip lock polythene bags that were well labeled, and transported to the laboratory. The samples were milled to a fine texture with a milling machine (Retsch, Model ZM200, Germany), packaged in sealed sterile plastic bags, and stored in a freezer at 4°C to avoid contamination. After each milling practice, 70% methanol was used to clean and decontaminate the equipment.

### 2.4 Sample preparation and cleanup for LC-MS/MS multi-mycotoxin analyses

Sample preparation and cleaning were done according to Berthiller et al. (2007) with 5 g of milled maize placed into a 50 mL tube (Sarstedt, Nümbrecht, Germany) and extracted for 90 min at 180 rpm on a rotary shaker (3017 GFL, Burgwedel, Germany) with 20 mL of extraction solvent (acetonitrile/water/acetic acid 79:20:1, v/v/v). The extracts were centrifuged for 2 min at 3,000 rpm on a GS-6 centrifuge (Beckman Coulter Inc., Fullerton, CA, USA). The raw extracts were transferred into glass vials using Pasteur pipettes, and 350 µL aliquots were diluted in the same volume (1/1) with dilution solvent, (acetonitrile/water/acetic acid 20:79:1, v/v/v) to adjust the solvent strength. After appropriate mixing, 50 µL of the diluted extract was analyzed using LC-MS/MS.

### 2.5 Method validation

The LC-MS/MS multi-mycotoxin method was validated in terms of linearity, apparent recovery (AP), limit of detection (LOD), and limit of quantification (LOQ), using the blank matrices of maize (401/2006/EC, 2006). The apparent recoveries of the analytes were calculated by spiking five different samples that were not contaminated with mycotoxins with a multi-analyte standard. The spiked samples (0.25 g each) were left overnight in the dark at room temperature to evaporate the solvent to establish equilibrium between the analytes. The sample was then extracted with 1 mL of extraction solvent as described above. The corresponding peak areas of the spiked samples were used to estimate the apparent recovery by comparison with a standard of the same concentration prepared by dilution in a pure solvent:


RA=100×(Peak area spiked samples)/(Peak area liquid standards)

LOD and LOQ were calculated from the signal-to-noise ratios (S/N) of the respective multiple reaction monitoring (MRM) chromatograms derived from the analysis of the spiked samples: LOD = 3 x S/N and LOQ = 10 x S/N, respectively.

### 2.6 LC-MS/MS multi-mycotoxin measurement parameters

The mycotoxin analyses were performed using the LC-MS/MS multi-mycotoxin method at the Centre for Analytical Chemistry, Department of Agrobiotechnology (IFA-Tulln), University of Natural Resources and Life Sciences, Vienna, Austria. The analyses were performed according to the methods described by Berthiller et al. (2007) and Malachová et al. (2018) with slight modifications.

A QTrap 4000 LC-MS/MS System (Applied Biosystems, Foster City, CA) was equipped with a turbo ion spray electrospray ionization (ESI) source and a 1100 Series HPLC System (Agilent, Waldbronn, Germany). Chromatographic separation of the analytes was done at 25°C on a Gemini^®^ C18 column, with 150 x 4.6 mm i.d. and 5 µm particle size, equipped with a C18 4 x 3 mm i.d. security guard cartridge (Phenomenex, Torrance, CA, US), using eluent A [methanol/water/acetic acid 10:89:1 (v/v/v)] or eluent B (methanol/water/acetic acid 97:2:1). Both eluents contained 5 mM ammonium acetate. After an initial time of 2 min at 100% A, the proportion of B was increased linearly to 100% within 12 min, followed by a hold-time of 3 min at 100% B and 4 min column re-equilibration at 100% A. The injection volume of 50 µL and flow rate of 1 mL min^-1^ was used. The ESI-MS/MS source temperature operated at 550°C, in the MRM mode, both in positive and negative polarities in two separate chromatographic runs per sample by scanning two fragmentation reactions per analyte. Further MS parameters were as follows: curtain gas 10 psi (69 kPa of max., 99.5% nitrogen); ion source gas 1 (sheath gas) 50 psi (345 kPa of nitrogen); ion source gas 2 (drying gas) 50 psi (345 kPa of nitrogen); ion spray voltage of -4000 V and +4000 V, respectively, collision-activated dissociation gas (nitrogen) high.

### 2.7 Data analysis

Microsoft Office Excel 2016 was used for the statistical analyses such as the mean and standard deviation of the different mycotoxins and to create different charts to visually compare the contamination values of the different mycotoxins.

## 3 Results and discussion

### 3.1 Summary of multi-mycotoxin occurrence in maize

The major mycotoxins found in South African commercial maize were aflatoxins (B_1_, B_2_, G_1_, and aflatoxicol), fumonisins (B_1_, B_2_, B_3_, B_4_, A_2_, and hydrolyzed B_2_), deoxynivalenol, DON-3-glucoside, 15-acetyldeoxynivalenol, 3-acetyldeoxynivalenol, nivalenol, zearalenone, zearalenone-sulfate, alpha-zearalenol, beta-zearalenol, and citrinin (CIT). No T2-toxin, HT-toxin, or ochratoxin A (OTA) were found in the post-harvest maize samples collected over the three production seasons. **Table 1** presents a summary of the occurrence of these mycotoxins and their mean values, maximum concentration values, and the rate of contamination.

**Table 1:** Multi-mycotoxin occurrence and mean and maximum concentrations in SA commercial maize samples.

Mycotoxin	Apparent recovery (%)	LOD (ppb)	LOQ (ppb)	Number of samples contaminated (n)	Contamination mean (ppb)	Range of contamination (ppb)	Percent Contamination (%)
Aflatoxin B_1_	58.2	0.22	0.72	4	6.45	0.22-19.77	0.53
Aflatoxin B_2_	60.8	0.06	0.21	2	1.32	0.06-2.22	0.27
Aflatoxin G_1_	84.7	0.16	0.54	3	4.23	0.16-7.46	0.4
Aflatoxicol	79.3	0.23	0.76	1	1.59	0.23-1.59	0.13
Fumonisin B_1_	90	2.4	8	372	237.31	2.4-7,373.33	49.47
Fumonisin B_2_	90	2.1	7	273	122.4	2.1-3,367.11	36.3
Fumonisin B_3_	90	2.1	7	163	56.78	2.1-619.20	21.68
Fumonisin B_4_	90	2.1	7	187	48.95	2.1-800.62	24.87
Fumonisin A_2_	90	2.4	8	29	20.61	2.4-40.74	3.86
Hydrolyzed fumonisin B_1_	104.9	0.2	0.68	27	2.81	0.2-10.76	3.59
Deoxynivalenol	80	1	8	656	542.54	1-13,320.00	87.47
DON-3-glucoside	101.7	0.75	2.5	621	60.17	0.75-1,574.83	82.58
15-ADON	89.2	9	30	559	161.4	9-2,450.22	74.34
3-ADON	64.1	4.8	16	46	33.4	4.8-134.17	6.12
Nivalenol	72.9	0.8	2.6	47	18.37	0.8-128.07	6.25
Zearalenone	85	0.2	0.6	396	20.49	0.2-593.69	52.66
Zearalenone-sulfate	99	0.2	0.7	402	43.73	0.2-623.03	53.46
Alpha-zearalenone	71.1	1.13	3.75	5	5.54	1.13-7.07	0.66
Beta-zearalenone	71.1	2.9	10	4	30.45	2.9-60.40	0.53
Citrinin	27.7	0.75	2.5	2	6.51	0.75-6.60	0.27

**Figure 2:**
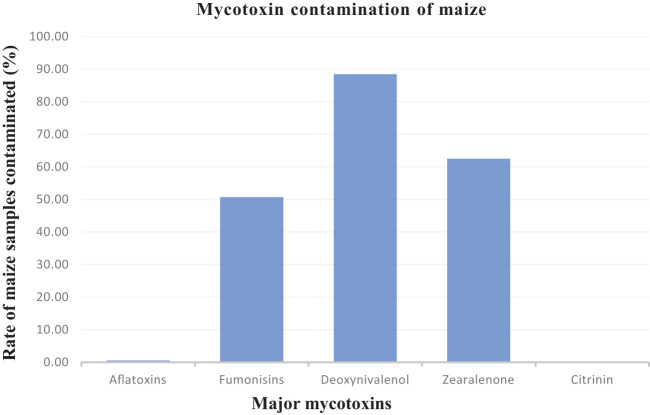
Mycotoxin contamination rates in maize samples.

For better presentation and discussion purposes, the above mycotoxins were further grouped into five main categories, for instance, aflatoxin B_1_, aflatoxin B_2_, aflatoxin G_1_, and aflatoxicol were grouped as total aflatoxins. This was repeated for total fumonisins, etc. Deoxynivalenol mycotoxins contaminated 665 samples, zearalenones 470 samples, fumonisins 381 samples, aflatoxins 4 samples, and the mycotoxin found in the lowest number of samples was citrinin which contaminated just two maize samples. **Figure 2** shows the rate of contamination of these categories of mycotoxins in the maize samples.

**Table 2** summarizes the different major mycotoxins that were isolated from the maize samples and shows their concentrations compared to SA and European Union (EU) regulatory limits. Aflatoxins, although only contaminating 0.53% of the samples, of these, 50% had concentrations above both the SA and EU limits. Deoxynivalenol contaminated 88.3% of the maize samples, of which, 31.33% had concentration levels above the EU limit and 4.82% above the SA limit. Furthermore, 62.5% of the maize samples were contaminated with zearalenone (ZEA) with none having concentrations above the SA limit and almost 2% with concentrations above the EU limit. Over 50% of the maize samples were contaminated with fumonisins with less than 10% having a concentration above the EU limit and 1% above the SA limit.

**Table 2:** Percentage of samples exceeding the EU and SA mycotoxin limits.

Mycotoxin	SA regulatory limit (ppb)	EU regulatory limit (ppb)	Positive samples (n)	Concentration (%)	Positive samples above the EU limit in all foods		Positive samples above the SA limit in all foods		Mean	Min. Value	Max. Value	STDV
					n	%	n	%				
Trichothecenes	2,000	500	664	88.3	208	31	32	4.82	738.78	3.44	17,488.7	1,103.447
ZEA	3,000 – 5,000	3,000	470	62.5	9	1.9	0	0	51.64	0.65	1,242.95	110.36
FBs	4,000	1,000	381	50.66	37	9.7	4	1.05	369.26	8.02	12,201.4	846.931
AFs	10	4	4	0.53	2	50	2	50	10.28	0.82	24.77	10.782
CIT	N/A	100	2	0.27	0	0	N/A	N/A	6.51	6.42	6.6	0.01414

### 3.2 Trichothecene mycotoxin (DON, DON-3-glucoside, 15-ADON, 3-ADON and nivalenol) prevalence in the maize samples

**Figure 3:**
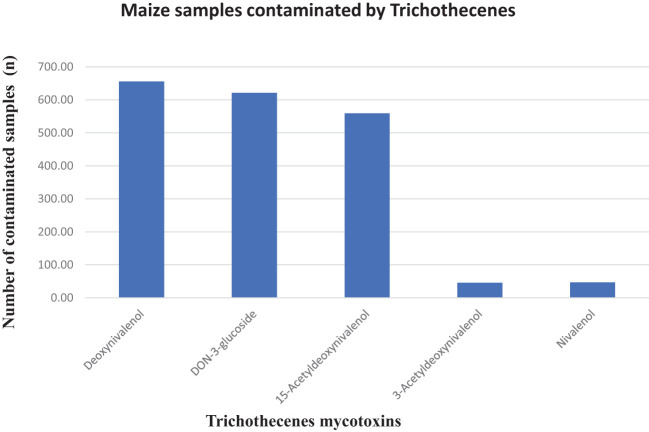
Trichothecene mycotoxins in maize samples.

Deoxynivalenol occurred in approximately 88% of the samples with a mean concentration of 542.54 ppb (max. 13,320 ppb). Similarly, DON-3-glucoside was identified in approximately 83% of the samples with a mean concentration of 61.17 ppb (max. 1,574.83 ppb), 15-acetyl-DON contaminated 74% of the samples with a mean concentration of 161.40 ppb (max. 2,450.22 ppb), and 3-acetyl-DON and nivalenol contaminated only approximately 6% of the collected maize samples with mean concentration values of 33.40 ppb (max. 134.17 ppb) and 18.37 ppb (max. 128.07 ppb) (**Table 1**). Maize in the field is naturally infected with the *Fusarium* species and geographic location is a determining factor. For instance, 3-acetyl-DON has been reported to be produced by *Fusarium graminearum* strains in Europe and Japan, and 15-acetyl-DON by North American strains (Perkowski et al., 1990), yet these two acetylated DON isomers were also present in the SA maize samples (**Figure 3**). DON was commonly only reported in samples originating from temperate regions (northern Europe and North America), but not anymore as reports from tropical countries (such as South Africa) continue to disclose the occurrence of DON in maize and maize products (Ekwomadu et al., 2020; Meyer et al., 2019; Shephard et al., 2011). In this study, approximately 88% of the analyzed samples contained DON with a maximum level of 13,320 ppb and 5% of the samples exceeded the maximum allowable level for DON in unprocessed maize of 2,000 ppb as set by South African regulations (**Table 2**). This work corroborates the 80.6% DON incidence rate reported in a Biomin study (2014–2017) that found that 9.76% of maize samples exceeded the EU regulatory limit (Gruber-Dorninger et al., 2018), as compared with 31.33% in this study (**Table 2**). DON is reportedly one of the most detected mycotoxins in cereal crops, and, depending on the concentration ingested, DON can be an immunosuppressant or stimulate the immune system to cause nausea and vomiting through interactions with the neural dopaminergic system and thus is also referred to as vomitoxin (Xu et al., 2020).

### 3.3 Fuminisin (B_1_, B_2_, B_3_, B_4_ and A_2_) prevalence in maize samples

**Figure 4:**
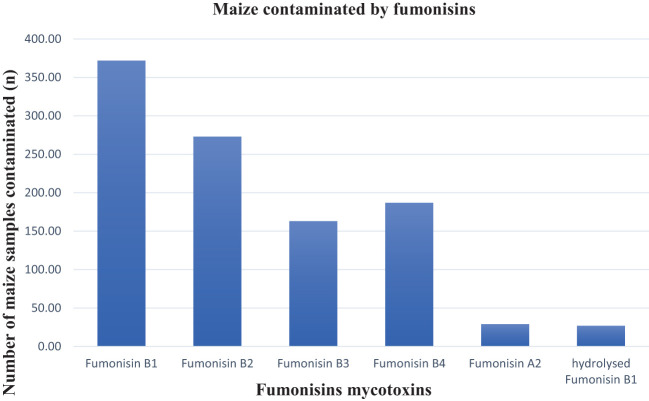
Types of fumonisins found in the maize samples.

Fumonisins are the common contaminant of SA maize. In this study, fumonisins B_1_, B_2_, B_3_, B_4_, and A_2_ were more frequently detected at higher levels in the maize samples compared to the other mycotoxins (**Table 1**). Fumonisin B_1_ was the most prevalent fumonisin contaminant, occurring in 49% of the samples (mean 237.31 ppb and max. 7,373.33 ppb), followed by fumonisin B_2_ in 36.3% (mean 122.4 ppb and max. 3,367.11 ppb), fumonisin B_4_ in 24.87% (mean 48.95 ppb and max. 800.62 ppb), fumonisin B_3_ in 21.68% (mean 56.78 ppb and max. 619.20 ppb), and, at 3.86% (mean 20,61ppb and max 40,74 ppb), fumonisin A_2_ contaminated the least number of samples (**Figure 4**). This can be attributed to the ability of *Fusarium* fungi to produce a high number of mycotoxins in warmer climates (Ekwomadu et al., 2020). Considering the fumonisins in total, approximately 51% of the samples were contaminated with at least one type of fumonisin, the maximum level of total fumonisins was 12,201.40 ppb and only 1% of the samples exceeded the maximum allowable levels in unprocessed maize of 4,000 ppb as set by South African regulations (**Table 2**). Compared with the 80.1% fumonisin incidence rate reported in the Biomin study (2014–2017), and, unlike the 1.3% of maize samples that exceeded the EU regulatory limit in that study (Gruber-Dorninger et al., 2018), in this study, over 9.7% of the samples exceeded the EU regulatory limit (**Table 2**). Some of the toxigenic health effects of fumonisins were well-reported by Ekwomadu et al. (2021). For example, fumonisin B1 has been implicated in human esophageal cancer in South Africa, China, and northeast Italy and is reported to be related to neural tube defects in the babies of mothers who consumed fumonisin-contaminated maize near the Texas–Mexico boundary (Ekwomadu et al., 2021; Missmer et al., 2006). All these circumstances are associated with the ingestion of fumonisin-contaminated maize. Impaired growth in children has been linked to a chronic intake of fumonisins (Kimanya et al., 2010).

### 3.4 Zearalenone, zearalenone-sulfate, alpha-zearalenone, and beta-zearalenone prevalence in maize samples

**Figure 5:**
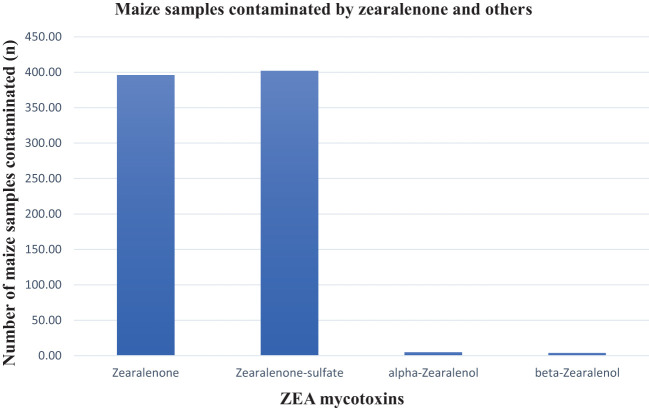
Zearalenone and associated mycotoxins in maize samples.

Overall, 62% of the samples were contaminated with zearalenone and its metabolites. Zearalenone and zearalenone-sulfate were observed in close to 53% (ZEA mean 20.49 ppb and max. 593,69 ppb; ZEA-sulfate mean 43.73 ppb and max. 623.03 ppb) of the maize samples. The prevalence of both alpha-zearalenone and beta-zearalenone was less than 1% in the maize samples with mean and maximum values of 5.54 ppb and 7.07 ppb, and 30.45 ppb and 60.40 ppb, respectively (**Table 1**). **Figure 5** presents a detail of their prevalences. These results corroborate the 47.1% zearalenone incidence rate reported in the Biomin study (2014–2017) and 3.8% of those maize samples exceeded the EU regulatory limit (Gruber-Dorninger et al., 2018). In this study, just 1.91% of samples exceeded the EU regulatory limits. None of the samples exceeded the maximum allowable levels for ZEA in unprocessed maize of between 3,000 and 5,000 ppb as set by the South African regulations (**Table 2**). Some of the properties of ZEA that have been reported include being estrogenic in humans as well as being immunotoxic, hepatotoxic, hematotoxic, and genotoxic, which may be partially attributed to ZEA contributing to oxidative DNA damage and cellular apoptosis (Waseem et al., 2014).

### 3.5 Prevalence of other mycotoxins in maize samples

**Figure 6:**
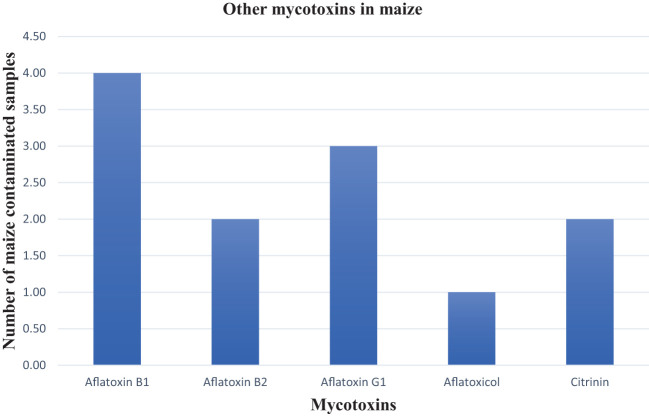
Other major mycotoxins in maize samples.

The aflatoxins AFB_1_, AFB_2_, AFG_1_, and aflatoxicol were reported in four maize samples. One sample was contaminated with 19.77 ppb AFB_1_, which was the highest contamination level of a single aflatoxin while the highest total aflatoxin contamination in a sample was 22.21 ppb (**Figure 6**). The aflatoxin findings in this study correlate with those reported by Meyer et al. (2019) and Gruber-Dorninger et al. (2018). Furthermore, this is the first time citrinin has been reported in SA maize with a maximum value of 6.60 ppb. No AFG_2_, T2-toxin, HT-toxin, or OTA were found in the post-harvest maize samples collected over the three production seasons. The concentration levels of citrinin were far below the EU regulatory limit of 100 ppb (EU 2019/1901).

Overall, DONs had the highest percentage of mycotoxin contamination (88%) of the maize samples in this study, of which approximately 5% were above the SA regulatory limit, followed by ZEAs (62.50%) but none of the samples had concentrations above the SA regulatory limit. Slightly above half the samples (50.66%) were contaminated with FBs and only 1% of the maize samples were contaminated with FB concentrations above the SA regulatory limit. This study corroborates previous studies carried out on SA commercial maize that predominantly found DONs, ZEAs, and FBs and did not find T2-toxin, HT-toxin, or OTA (Ekwomadu et al., 2020; Gruber-Dorninger et al., 2018; Meyer et al., 2019). *Fusarium* mycotoxins have always been an issue in South African maize as incidences of human esophageal cancer were reported in 1990 to be associated with fumonisin B1 (Nji et al., 2022d). This is the first time that citrinin has been detected in South African commercial maize. Citrinin is a secondary metabolite produced by some fungal strains such as the *Aspergillus*, *Penicillium*, and *Monascus* species (Zargar and Wani, 2023). Consumption of citrinin-contaminated food and feed by both humans and animals has been linked to serious health concerns across the globe. Citrinin affects all the main organs, including the bone marrow, liver, kidney, and mitochondrial respiratory chain and has been linked to human genotoxic, embryotoxic, teratogenic, carcinogenic, and mycotoxin nephropathy consequences (Chai et al., 2020). As mentioned previously, mycotoxins of concern in the sub-Saharan Africa include AFs, OT, FBs, DON, ZEAs, patulin (PAT), and CIT (Alshannaq and Yu, 2017; Martínez-Culebras et al., 2021; Mogopodi et al., 2022). In this study, multi-mycotoxin contamination of South African commercial maize was analyzed and almost all the above listed mycotoxins (ZEAs, FBs, DON, AFs, and CIT) of concern were isolated to varying degrees. For instance, less than 1% of the 752 maize samples were contaminated by both aflatoxins and citrinin. CIT has been shown to be nephrotoxic in all tested animal species (Xu et al., 2020). Although the mean value of total aflatoxin in this study was above both the EU and the SA regulatory values, only four samples were contaminated. The rest of the mycotoxins had mean values within the acceptable limits for both the EU and SA regulatory limits.

## 4 Conclusion

In this study, a multi-mycotoxin analysis of maize samples over a 3-year period was conducted. At least 88% of the maize samples were contaminated with one or more mycotoxins, with *Fusarium* mycotoxins contaminating the most. Amongst the mycotoxins reported to be of major public health and agro-economic concern, all were quantified in this study except for ochratoxin and patulin. In this study, citrinin was reported in South African commercial maize for the first time. Less than 5% of the maize samples were contaminated by mycotoxins above the SA regulatory limit. Aflatoxin contamination of maize is not an issue in South Africa. Continuous monitoring and evaluation of SA commercial maize for multi-mycotoxin analysis is recommended, especially with the ongoing global warming and climate change, to provide the necessary data in order for legislative bodies to make informed decisions.

## Data Availability

The original contributions presented in the study are included in the article/**Supplementary Material**. Further inquiries can be directed to the corresponding author.
